# Leukocyte Heparanase: A Double-Edged Sword in Tumor Progression

**DOI:** 10.3389/fonc.2019.00331

**Published:** 2019-04-29

**Authors:** Alyce J. Mayfosh, Nikola Baschuk, Mark D. Hulett

**Affiliations:** ^1^Department of Biochemistry and Genetics, La Trobe Institute for Molecular Science, La Trobe University, Melbourne, VIC, Australia; ^2^Centre for Innate Immunity and Infectious Diseases, Hudson Institute of Medical Research, Melbourne, VIC, Australia

**Keywords:** heparanase, leukocytes, macrophages, natural killer cells, immunotherapy, tumor progression

## Abstract

Heparanase is a β-D-endoglucuronidase that cleaves heparan sulfate, a complex glycosaminoglycan found ubiquitously throughout mammalian cells and tissues. Heparanase has been strongly associated with important pathological processes including inflammatory disease and tumor metastasis, through its ability to promote various cellular functions such as cell migration, invasion, adhesion, and cytokine release. A number of cell types express heparanase including leukocytes, cells of the vasculature as well as tumor cells. However, the relative contribution of heparanase from these different cell sources to these processes is poorly defined. It is now well-established that the immune system plays a critical role in shaping tumor progression. Intriguingly, leukocyte-derived heparanase has been shown to either assist or impede tumor progression, depending on the setting. This review covers our current knowledge of heparanase in immune regulation of tumor progression, as well as the potential applications and implications of exploiting or inhibiting heparanase in cancer therapy.

## Introduction

Heparanase is the only mammalian enzyme that directly cleaves heparan sulfate side chains of heparan sulfate proteoglycans (HSPGs), key components of the extracellular matrix and basement membrane. The cleavage of heparan sulfate by heparanase regulates a number of fundamental cellular processes including cell migration (1, 2), cytokine production (3, 4), angiogenesis (5), and wound healing (6). Furthermore, heparanase has also been implicated in cell adhesion that is independent of its enzymatic activity (7, 8). The ability of heparanase to regulate these processes also makes it a key player in several pathological settings such as inflammatory disease and cancer. Heparanase contributes to various inflammatory diseases including delayed hypersensitivity, vascular injury, chronic colitis, Crohn's disease, sepsis, rheumatoid arthritis (9), atherosclerosis (10), and diabetes (11–13). Furthermore, heparanase is upregulated in response to pro-inflammatory cytokines, bacterial or viral infections, and modulates innate immune cell function. For example, in sepsis heparanase is upregulated by tumor necrosis factor-α (TNF-α) and induces shedding of the glycocalyx, thereby exposing the endothelial surface and adhesion molecules which facilitate neutrophil recruitment (14). Heparanase has also been well-characterized in cancer (15, 16), where the overexpression of heparanase often contributes to tumor progression (17, 18). The overexpression of heparanase has been detected in almost all cancer types, where it promotes metastasis (19–21), angiogenesis (19, 21, 22), and tumor proliferation (23). More recently, the role of leukocyte heparanase in tumor progression has been more closely examined, with the suggestion that it can be either pro- or anti-tumorigenic, depending on the setting.

## Heparanase Expression by Leukocytes

The first documentation of heparanase expression in leukocytes was in T lymphocytes where the production of an endoglycosidase was observed in assisting their migration and penetration of the basement membrane and blood vessel entry (24). Subsequently, heparanase expression has been further characterized in T cells (25–30) as well as a number of other leukocytes including B cells (31), natural killer (NK) cells (2), monocytes (32), dendritic cells (DCs) (1, 32), macrophages (29, 30, 33–37), neutrophils (38–40), mast cells (41), and eosinophils (42). The expression of heparanase by leukocytes is inducible by various cell activatory stimuli (2, 43, 44) and has been shown to promote leukocyte migration (1, 45), cell rolling and adhesion (46, 47), the upregulation of pro-inflammatory cytokines (3), and activation of innate immune cells (34). Heparanase has also been associated with inflammatory diseases such as atherosclerosis (10) and diabetes (11–13). However, despite this progress, much remains to fully understand the role of heparanase in leukocytes and its contribution to disease. It is well-established that leukocytes are important regulators of tumor progression (48–51). An emerging area of significant clinical interest is at the intersection of heparanase, leukocytes, and cancer. We will now discuss how heparanase may regulate leukocyte function in the context of tumor progression and its relevance in cancer therapy.

## Leukocyte Heparanase and Tumor Progression

### Heparanase and Macrophage Activation and Infiltration Into Tumors

**Figure 1:**
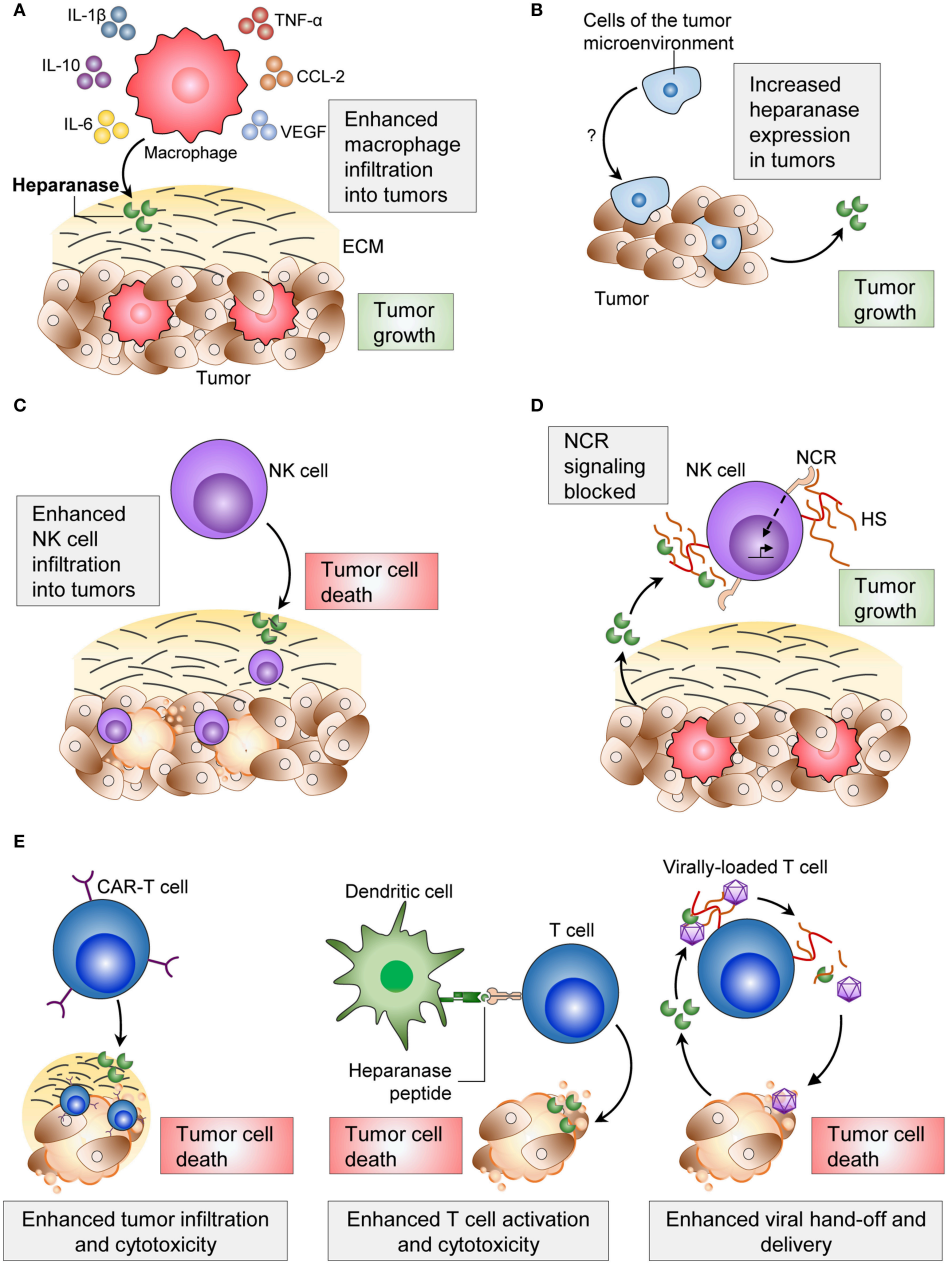
Effects of heparanase on immune cells and the consequences on tumor progression. **(A)** Heparanase from macrophages and from tumor cells increase macrophage infiltration into tumors, cytokine secretion and phagocytic ability. **(B)** Cells of the tumor microenvironment increase tumor cell-heparanase and increase tumor cell proliferation. **(C)** Heparanase enhances NK cell infiltration into tumors and consequent tumor cell clearance. **(D)** Tumor cell-heparanase can block NCR signaling and consequent activation of NK cells. **(E)** Applications of immune cell-heparanase include use in CAR T cells, dendritic cell vaccines, and viral delivery of anti-tumorigenic agents. ECM, extracellular matrix; NCR, natural killer cytotoxicity receptor; HS, heparan sulfate; CAR-T, chimeric antigen receptor-T.

Tumor associated macrophages (TAMs) are often found within primary tumors and pre-metastatic sites, and their presence frequently contributes to tumor progression (52, 53). In heparanase knockout mice, macrophage infiltration into implanted Lewis lung carcinoma tumors was impaired, and tumors were smaller than in wild type animals (34). Macrophages from heparanase knockout mice also expressed lower levels of the pro-inflammatory cytokines TNF-α, interleukin-1 β (IL-1β), C-X-C motif chemokine ligand 2 (CXCL2) and IL-6 (34, 54). The opposite was observed in a model of pancreatic cancer overexpressing heparanase. Pancreatic tumor cells overexpressing heparanase were implanted into severe combined immune deficiency (SCID) mice, which lack B and T cells (55). Implanted tumors with heparanase-overexpressing pancreatic cancer cells were observed to have more infiltrating macrophages and larger tumors compared to tumors with normal heparanase expression (54) (Figure 1A). The overexpression of heparanase in these pancreatic tumors also led to increased macrophage expression of IL-6, IL-10, C-C motif chemokine ligand-2 (CCL-2), vascular endothelial growth factor (VEGF) and macrophage scavenger receptor-2 (MSR-2) (54) (Figure 1A). Indeed, TAM expression of these cytokines is an indicator of macrophage polarization to an M2 phenotype, which facilitates tumorigenesis (52, 56, 57). These findings suggest that both tumor-derived and macrophage-derived heparanase can promote the recruitment of macrophages to tumors and facilitate their entry to aid tumor progression.

During inflammation and inflammation-associated tumorigenesis, the source of heparanase is often the epithelium (58). This was identified in patients with inflammatory bowel disease (IBD) (59), and in an IBD model, epithelial cell-heparanase was found to drive the over-activation of macrophages, inflammation, and ultimately tumorigenesis (60). In this model of IBD, heparanase-overexpressing mice were also observed to have more macrophages in the colon when compared to wild type animals (60). This overexpression of heparanase in the epithelium has been characterized in other models of inflammation, including pancreatitis (61) and Barrett's epithelium in the esophagus (62). However, it remains to be explored whether epithelial cell-heparanase in these settings also influences immune cell activation. Furthermore, another study found that recombinant heparanase added to colorectal cancer cell lines could increase mRNA expression and release of monocyte chemoattractant protein-1 (MCP-1) (63), supporting the notion that heparanase may help to generate a chemokine gradient to recruit macrophages to sites of inflammation.

Together, these findings show that heparanase from the tumor cells, macrophages, and epithelial cells can promote tumorigenesis. However, not all tumor cells overexpress heparanase. Weissmann et al. found that Raji lymphoma cells expressed low levels of heparanase *in vitro*, but when implanted into mice, exhibited increased heparanase activity (22) (Figure 1B). This may have been a result of the tumor cells upregulating heparanase in response to stimuli from the tumor microenvironment, which could include soluble factors such as TNF-α and IL-1β (35, 43), or heparanase may have originated from other cell types within the tumor microenvironment (e.g., macrophages). Regardless of the source of heparanase, its inhibition with the heparanase neutralizing antibody Ab 1453 in these tumors was sufficient to reduce tumor growth (22). Again, the source of heparanase in this example is unclear, but this study supports the idea that tumor cells can utilize heparanase from the tumor microenvironment with similar outcomes on tumor progression.

### Tumor Cells Modulate Heparanase Expression in Lymphocytes

Tumor cells can influence leukocyte function via direct cell-cell interaction (64), or through secreted factors (65). A study by Theodoro et al. found that lymphocytes from peripheral blood mononuclear cells (PBMCs) of breast cancer patients displayed higher heparanase expression than lymphocytes from healthy patients (66). The study also found that heparanase expression was higher in lymphocytes from patients with metastases, and that heparanase expression in circulating lymphocytes was reduced following surgical resection of tumors (66). Breast tumor cells when co-cultured with lymphocytes from healthy donors were shown to induce heparanase expression by the lymphocytes. Furthermore, these experiments suggested that the breast tumor cells induced the lymphocytes to produce soluble factors that were responsible for upregulating heparanase expression (66). It was proposed that by increasing expression of heparanase in tumor-infiltrating lymphocytes, the tumor would have the ability to alter gene expression of many other neoplastic and non-neoplastic cells (66). The impact of these high-heparanase expressing lymphocytes was not tested. However, since these patients had higher instances of metastasis, it suggests that these lymphocytes may be preparing to seek out tumor cells for clearance, given that heparanase is often upregulated upon T cell activation (24, 25, 27, 28).

### Heparanase and NK Cell-Mediated Clearance of Tumors

NK cells efficiently kill tumor cells of many origins, and their presence within tumors often correlates with improved survival (67). We recently reported that mice deficient in NK cell-heparanase exhibited reduced NK cell tumor infiltration, resulting in impaired clearance of B16F10 melanoma tumors and metastases (2) (Figure 1C). Furthermore, immune checkpoint inhibitors targeting the programmed death ligand-1 (PDL-1) and cytotoxic T-lymphocyte-associated antigen 4 (CTLA-4) axes were less effective in the absence of NK cell-heparanase (2). These data suggest that using heparanase inhibitors concomitantly with checkpoint inhibitors would be ineffective. To our knowledge, this is the first description of a tumor suppressive role for heparanase.

### Heparanase Blocks NK Cell Activation

The role of heparanase in NK cell function does not appear to be simply in cell migration and invasion, but may also regulate NK cell activation and cytotoxicity.

Heparan sulfate on the plasma membrane of NK cells can act in a *-cis* manner as a co-ligand for the NK cell cytotoxicity receptor (NCR) (68). However, surface heparan sulfate must compete with soluble or *-trans* heparan sulfate for NCR binding, which dampens NK cell activation. By cleaving heparan sulfate on the surface of NK cells, heparanase secreted by tumors can increase levels of soluble heparan sulfate, and consequently inhibit NK cell activity and cytotoxicity against tumor cells (69) (Figure 1D). It appears that low levels (69) or high levels (68) of *-cis* heparan sulfate-NCR interaction dampens NK cell activation, and that maintaining optimal levels of membrane-bound heparan sulfate is important for optimizing NK cell activation.

Despite these advances, further investigation is required to fully understand the role of heparanase in leukocyte function during cancer progression. Given the ability of heparanase to modulate pro-inflammatory cytokine levels (3, 60), and activate and recruit tumor-promoting leukocytes (34, 54), it is likely that heparanase plays a greater role in modulating the immune system and immune suppression during cancer progression.

## Exploiting Heparanase in Cancer Immunotherapy

Despite the complex pro/anti-tumorigenic axis of heparanase, exploiting heparanase has shown promise in leukocyte-based anti-cancer therapies.

### Heparanase in CAR-T Cell Therapy of Solid Tumors

Chimeric antigen receptor (CAR)-T cell therapy utilizes engineered recombinant receptors expressed on T cells containing an antigen-recognition domain of a monoclonal antibody and a T cell-activating domain (70). These CARs enable T cells to specifically and efficiently recognize tumor cells and maximize T cell function. Whilst this therapy has shown promise in many hematological malignancies (71, 72), it is relatively ineffective against solid tumors, partly attributed to the low penetration of CAR-T cells into the tumor (70). To address this, Caruana et al. overexpressed heparanase in human CAR-T cells, and found this to assist CAR-T cell infiltration into neuroblastoma patient-xenograft tumors and enhance anti-tumor activity (73) (Figure 1E). This strategy of using heparanase to increase the penetration of CAR-T cells into tumors shows promise to increase efficacy of the therapy.

### Heparanase in DC Vaccines

Heparanase overexpression has been documented across the majority of tumor types, including solid tumors (74–77) and hematological tumors (22, 78). Thus, heparanase represents a potential tumor associated antigen (TAA) that could be exploited across multiple cancer types. Dendritic cell vaccines are a novel approach to selectively target tumor cells overexpressing TAA. Engineered dendritic cells overexpressing TAA can generate antigen-specific T cells that have increased cytotoxicity against tumor cells (79–81).

Heparanase-specific and reactive CD8^+^ T cells were identified in the bone marrow of a sample of breast cancer patients, and were functionally reactive to heparanase-overexpressing tumor cells (82). The overexpression of heparanase in DCs isolated from PBMCs was shown to enhance the activation of T cells from matching donors, and consequent cytotoxicity against target gastric cancer cells (83). This finding also held true in an animal model, where murine DCs were pulsed with murine heparanase peptides and injected into mice. This vaccine could induce cytotoxic T lymphocytes (CTLs) in mice specific to H-2kb-expressing mouse tumor cell lines (B16, LLC, and EL-4) (84). In addition, administering heparanase peptide-pulsed DCs after injecting B16 tumor cells could slow tumor growth (84). Furthermore, the immunogenicity and efficacy of these peptides was increased when generated in the branched multiple antigenic peptide conformation (85, 86).

The heparanase peptide has also been tested as a TAA in a prophylactic vaccine. Priming mice *in vivo* with human heparanase peptides (Hpa525, Hpa277, and Hpa405) generated CTLs that specifically targeted human tumor cell lines presenting heparanase on either HLA-A^*^0201 (87) or HLA-A2 (88) (Figure 1E). Injecting heparanase-pulsed DCs into mice before administering B16 tumor cells was shown to protect animals from tumor growth (84).

These data suggest that heparanase could be a robust tumor antigen, as when targeted, shows both reduction and protection against tumor growth in animal and human systems. However, targeting heparanase via a vaccine approach will rely on tumors maintaining heparanase expression to allow T cell recognition of heparanase-positive tumor cells. Regardless, the selective pressure from these vaccines on tumor cells to downregulate heparanase expression would still be advantageous in blocking tumor progression.

### Heparanase in Viral-Therapeutic Delivery

The delivery of gene therapy as a cancer treatment requires specific targeting to tumor cells. A promising approach to target therapies toward tumors is through viral particles via attachment to T cells (89). An important step in the delivery of these therapies is the release or “hand off” (transfer of viral particles from T cells to target tumor cells), when T cells release their viral cargo at the tumor site (89). Heparanase present in the tumor microenvironment, either from malignant cells or activated T cells, was shown to promote viral “hand off” for the successful delivery of anti-tumor molecules to the tumor cells (89) (Figure 1E). Another study using viral gene therapy to treat a murine model of malignant plural mesothelioma found that co-infection with a heparanase-expressing adenovirus vector could enhance efficacy of virotherapy and penetrance into tumors (90), a previous limitation of this therapy. This approach showed a reduction in tumor weight, and an increase in overall survival of animals inoculated with mesothelioma. This is likely a result of heparanase increasing ECM breakdown, as heparanase was shown to enable viral particles to penetrate deeper into tumor spheroids (90).

The robust expression of heparanase across multiple cancer types and cell types makes it a useful target to manipulate and utilize its anti-cancer properties. All of these therapies described will rely on maintained heparanase expression for efficacy. As we will describe, heparanase inhibitors currently used to reduce tumor burden may not always have favorable outcomes on tumor progression, especially for patients undergoing the therapies described above.

## Heparanase Inhibitors and Leukocyte Function

Our understanding of the relationship between heparanase and leukocytes during tumor progression remains limited. Similarly, much is still unknown about how heparanase inhibitors affect the anti-tumor immune response, despite their current use in clinical trials against a range of cancers (91–94). A number of heparanase inhibitors in anticancer therapy have been recently reviewed (95–97). These include the heparan sulfate mimetic Roneparstat (SST0001), 2-O-,3-O-desulfated heparin (ODSH, also known as CX-01), Necuparanib (M402), PG545 (a heparan sulfate mimetic conjugated to a lipophilic cholestanol aglycone moiety, also known as Pixatimod) (21), and PI-88 (a heparan sulfate mimetic, also known as Muparfostat) (98).

Preclinical animal models show PG545 can reduce tumor and metastatic burden in several tumor models, including breast, prostate (21), liver (21), lung (21), colon (21), ovarian (94), head, and neck cancers (21, 99, 100), melanoma (21, 101), pancreatic cancer (102, 103), and colon cancer (104). Interestingly, the mechanism by which PG545 exerts its anti-tumor properties has been shown to be multifactorial. In addition to directly blocking heparanase activity (105), PG545 has been shown to reduce heparanase expression, possibly by inhibiting VEGF and FGF2 signaling (99). PG545 has also been shown to inhibit macrophage infiltration into pancreatic tumors (102), activate NK cells via DCs (106), and activate lymphocytes (100), as part of its anti-tumor activity. A phase I clinical trial against a range of advanced solid tumors showed that PG545 stimulated the innate immune response, resulting in an at least a two-fold increase of circulating plasmacytoid DCs and NK cells in majority of patients (93). It is perhaps predominantly through this mechanism of leukocyte activation that this inhibitor exerts its anti-cancer activity, rather than direct tumor cell-heparanase inhibition. Given its modest effects as a monotherapy (93), PG545 will most likely be used in combination with chemotherapy to treat advanced cancers for maximum efficacy.

Other clinically relevant heparanase inhibitors such as Roneparstat and ODSH also display immunomodulatory effects. Roneparstat, in development for the treatment of multiple myeloma, has been observed to effect macrophage polarization by inhibiting the expression of M1 related genes in LPS-stimulated U937 macrophages (107). In a mouse model of ischemia/reperfusion injury, inhibition of heparanase with Roneparstat reduced the number of infiltrating M1 macrophages in the kidney, resulting in lower levels of pro-inflammatory cytokines (107). ODSH is another heparanase inhibitor (108) which blocks multiple steps of inflammation. As described for heparin, ODSH reduces leukocyte rolling, adhesion, and accumulation (109, 110). ODSH has also been shown to inhibit neutrophil elastase and inflammation in a mouse model of neutrophil elastase-induced airway inflammation (111) and in the sputum of cystic fibrosis patients (112). In addition, ODSH also inhibits the accumulation of neutrophils in the airway after *Pseudomonas aeruginosa* infection (113) and protects against platelet factor 4-induced thrombocytopenia in chemotherapy and radiotherapy-treated animals by acting on megakaryocyte proliferation. Finally, ODSH inhibits high-mobility group box 1 (HMGB1) release from macrophages (111, 113, 114), a potent proinflammatory cytokine, and inhibits P-selectin-mediated macrophage adhesion (115).

More work needs to be done to define and understand the effects of heparanase inhibitors on cells of the immune system. Heparanase inhibitors have been used as anti-inflammatory agents, and have been shown to impair lymphocyte trafficking (116, 117) and leukocyte function (46, 47, 61, 118). It is possible that in some tumor settings, heparanase inhibitors may inhibit leukocyte function, and consequently tip the balance away from tumor clearance and in favor of tumor progression. Heparanase inhibitors may be effective against tumors in which leukocyte-heparanase aids tumor progression, such as colorectal and pancreatic carcinoma (52), but perhaps less effective against other solid tumors which have little heparanase expression in the tumor microenvironment. Choosing the appropriate anti-cancer therapy will lie in finding the balance in particular cancer settings between inhibiting pro-tumorigenic heparanase and promoting its anti-tumorigenic effects.

## Conclusions

This review describes how leukocyte-heparanase can be a double-edged sword in tumor progression; it can enhance tumor immune surveillance and tumor cell clearance, but also promote tumor survival and growth. We also discuss the potential of using heparanase in leukocyte therapies against tumors, and the effects of heparanase inhibitors on tumor progression and immunity.

We are just beginning to understand the influence of heparanase on a pro/anti-tumor immune response, and there are still many questions to answer. How do the pro/anti-tumorigenic effects of heparanase differ across different cancer types? Does the tumorigenic effect of heparanase change during cancer progression? And how does the expression or role of heparanase change during treatment regimens? Answering these questions may help guide the appropriate use of heparanase inhibitors, and the use of heparanase-assisted therapies for the treatment of cancer.

## Author Contributions

All authors listed have made a substantial, direct and intellectual contribution to the work, and approved it for publication.

### Conflict of Interest Statement

The authors declare that the research was conducted in the absence of any commercial or financial relationships that could be construed as a potential conflict of interest.
